# Inflammasome effects in GvHD

**DOI:** 10.18632/oncotarget.6307

**Published:** 2015-11-11

**Authors:** Brent H. Koehn, Robert Zeiser, Bruce R. Blazar

**Affiliations:** Department of Pediatrics, Division of Blood and Marrow Transplantation, University of Minnesota Cancer Center, Minneapolis, MN, USA

**Keywords:** GVHD, inflammasome, myeloid suppressor cells

Myeloid-derived suppressor cells (MDSCs) are a naturally occurring immune regulatory population capable of suppressing inflammation, and are often associated with cancer, chronic infection and traumatic injury [1]. Growing interest in the application of MDSC therapeutically has been supported by preclinical studies in allo-graft rejection, autoimmunity and GvHD. Our studies find that normal bone marrow can be used to generate MDSC and when activated by the cytokine IL-13 produce arginase-1, which enzymatically depletes L-arginine and contributes to T cell dysfunction/suppression, leading to a reduction but not elimination of GVHD lethality [2]. In a recent paper, we show that the destructive environment characteristic of GvHD mice causes a loss of MDSC suppressor function due to activation of the inflammasome, a multi-protein oligomer that results in caspase-1 activation and promotion of inflammation [3].

**Figure 1:**
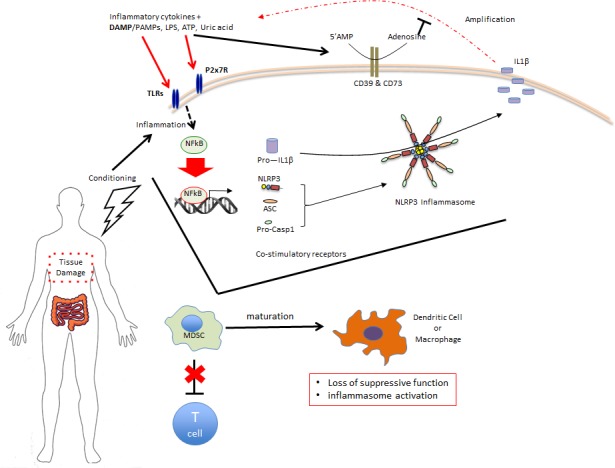
Conditioning associated with BMT generates tissue damage and inflammasome inciting factors which activate transferred MDSC thereby limiting functional activity

Activation of the inflammasome can be via endogenous damage-associated molecules termed DAMPs (Figure 1). We have shown that ATP is released from dying cells after preconditioning treatment prior to allo-HCT and that it serves as a critical danger signal for the activation of the immune system [4]. ATP binds to the purinergic P2X7 receptor on APCs and leads to increased expression of costimulatory molecules, followed by stronger activation of alloreactive T cells and a more severe GvHD phenotype [4]. The activation of purinergic receptors is tightly regulated by ecto-5′-nucleotidase/CD73 that catalyzes the dephosphorylation of extracellular nucleotide 5′-monophosphates. CD73 deficiency of donor or recipient leads to significantly enhanced GVHD with reduced survival of the recipient and increased GvHD histopathology scores [5]. P2X7 activation or exposure of myeloid cells to uric acid can activate the NLR, pyrin domain containing 3 (Nlrp3) component of inflammasome, relevant in the early phase of GvHD as mice deficiency in Nlrp3 or ASC results in less severe GVHD [6]. Recently a connection between miR-155 and inflammasome activation was detected in myeloid antigen presenting cells by using unbiased transcription as well as functional studies showing that Nlrp3/miR-155 double-knockout allo-HCT recipient mice had no increased protection from GVHD compared with Nlrp3 deficient recipients [7]. Collectively these data indicate that DAMPs may shape the phenotype of myeloid cells and thereby the decision if a cell becomes pro- and anti-inflammatory.

Myeloid cells are uniquely responsive to their environment and in our efforts to improve MDSC function, we investigated their inflammasome activation status both *in vitro* and in the context of GvHD [3]. In as little as five days post transplant transferred MDSC demonstrated an activated CD11c+ phenotype as well as hallmarks of inflammasome activation, including caspase-1 cleavage and secretion of IL-1β. Notably, *ex vivo* suppressive capacity of MDSC was also reduced. The temporal nature of MDSC loss of function was addressed by applying multiple consecutive doses of MDSC-IL13 (days 0, 3 & 6), resulting in enhanced overall survival, suggesting MDSC functionality is compromised early due to GvHD conditions and that maintaining a suppressive environment using repetitive MDSC infusions could overcome the loss of suppression of an individual MDSC infusion. In support of an inflammasome mediated loss of function, genetic ablation of the inflammasome-associated adaptor protein ASC (Apoptosis-associated speck-like protein containing a CARD) prevented MDSC inflammasome activation and resulted in improved survival relative to wild-type MDSC in GvHD mice. Moreover, ASC^−/−^ MDSC-IL13 recovered from day 5 post transplant recipients did not produce IL-1β and better retained their capacity to suppress T cell proliferation *ex vivo* than wild-type MDSC-IL13 recovered from GvHD mice. The translational potential of these findings are supported by the fact that we observed human peripheral blood-derived MDSC to be susceptible to *in vitro* inflammasome activation and had reduced suppressive function under these conditions.

Myeloid cells play a critical role in developing, shaping and sustaining immune responses and are remarkably adaptable to their environment. Therefore, adoptive cell therapies using MDSC need to be tailored and monitored carefully, as culture conditions are vastly different then the conditions MDSC are exposed to upon transfer. An important question that remains is elucidating which inciting factors are involved in transplant/GvHD associated inflammasome activation. While ATP/P2x7R and Nlrp3/miR-155 are recognized as important targets with relevance in GvHD, a wide variety of factors are known to activate inflammasomes including DAMPs, bacterial & viral (dsDNA) products and environmental stimuli (alum, uric acid) and may contribute to MDSC inflammasome activation. Whether it is possible or necessary to globally inhibit these upstream pathways for inflammasome activation is yet unclear. Our future aims are to produce higher functioning MDSC that are capable of sustained function in the harsh early post transplant setting of GvHD. Methods for tailoring MDSC to resist conversion include genetic alterations to prevent inflammasome activation, as seen using ASC^−/−^ MDSC, and clinically viable options including mRNA or gene knockdown or knockout technologies such as sh/siRNA or nucleases that target one or more inflammasome components. Pharmacologic agents such as small molecule inhibitors designed to repress inflammasomes currently being developed to circumvent GvHD pathology are expected to act on transferred MDSC as well, and we would predict aid in maintenance of function. Other implications from this work include the potential to conversely promote inflammasome activation locally at the site of tumor burden for the purposeful enhancement of immune activation and anti-tumor immune therapy, as MDSC are nearly ubiquitously observed in the setting of solid tumors. Our findings suggest that the degree of inflammation is critical for MDSC functional support, and anti-tumor therapies may benefit from local or systemic priming of inflammasome activation, converting tumor-associated MDSC and releasing their suppressive potential.
